# Use of telemedicine in the outpatient sector during the COVID-19 pandemic: a cross-sectional survey of German physicians

**DOI:** 10.1186/s12875-022-01699-7

**Published:** 2022-04-23

**Authors:** Vera Knörr, Lorena Dini, Sophie Gunkel, Jan Hoffmann, Laura Mause, Tim Ohnhäuser, Arno Stöcker, Nadine Scholten

**Affiliations:** 1 Institute of Medical Sociology, Health Services Research, and Rehabilitation Science, Faculty of Human Sciences, Faculty of Medicine at the University Hospital, University of Cologne, Cologne, Germany; 2 Institute of General Practice, Charité – Universitätsmedizin Berlin, Berlin, Germany

**Keywords:** COVID-19, Pandemics, Telemedicine, Telephone consultation, Video consultation, General medicine

## Abstract

**Background:**

In the wake of the coronavirus disease 2019 (COVID-19) pandemic, administrative barriers to the use of telemedicine have been reduced in Germany. The study focused on the analysis of use and assessment of telemedicine by physicians working in the outpatient sector, considering the perspective of different disciplines during the COVID-19 pandemic in Germany.

**Methods:**

The anonymous cross-sectional online survey within the study COVID-GAMS was conducted from 16 November 2020 to 1 January 2021. General practitioners; cardiologists; gastroenterologists; paediatricians; gynaecologists; ear, nose, and throat (ENT) specialists were randomly selected and invited to participate in the survey. At the same time, open recruitment to the online survey was conducted via the professional societies. Descriptive and regression analyses were performed based on the data of 1521 outpatient responding physicians.

**Results:**

The use of telephone and video consultation increased during the pandemic. Regarding the frequency of use, physicians already using telephone/video consultations in March/April 2020 report an increase in such services. General medicine was associated with an increased use of telephone and video consultations than cardiology, gynaecology or ENT, and in the case of telephone consultations also compared to paediatrics. General practitioners assessed the subjective usefulness higher than gynaecology and ENT. And the self-reported proportion of patients receiving telemedical care was higher correlated with general medicine than all other disciplines. The location of the practice (rural vs. urban), type of practice (individual vs. group) and gender (male vs. female) were also shown to be significant influencing factors on the variables mentioned above. Barriers reported by physicians not using telemedicine were the lack of equivalence to face-to-face contact and perceived low demand from patients**.**

**Conclusion:**

The COVID-19 pandemic has led to a significant increase in the use of telemedicine, to varying degrees in the different specialities. Individual and structural factors lead to a reduced use of telemedicine and there are physician’s and patient’s barriers that have prevented telephone and video consultations from gaining acceptance by physicians. All these factors must be addressed if telemedicine procedures are to be implemented widely.

**Supplementary Information:**

The online version contains supplementary material available at 10.1186/s12875-022-01699-7.

## Background

Telemedicine can be defined as a collective term for various medical care concepts. In this context, information and communication technologies (ICT) are used to provide medical services to the population in the areas of diagnostics, therapy and rehabilitation as well as medical decision-making advice across spatial distances [1]. ICTs, such as telephone or video consultations, enable physician–patient communication without direct physical contact. The lack of direct physical contact, which is associated with the risk of possible infection, is particularly relevant in the context of the pandemic [2]. In the past, disasters, epidemics, and pandemics have often led to advances in innovative techniques, such as telemedicine [3, 4]. The coronavirus disease 2019 (COVID-19) pandemic has accelerated the process of implementing this technical innovation internationally, as well as in Germany, as the use of telemedicine procedures became necessary in many places owing to the existing contact restrictions [5–11].

Before the outbreak of the COVID-19 pandemic, telemedicine procedures were little used in Germany and their integration into everyday medical practice was extremely low by international standards [12]. The restriction on video consultation hours for physicians and psychotherapists was suspended at the beginning of April 2020. Since 1 October 2020, the field of use for case conferences and case discussions via video calling has been expanded, and the National Association of Statutory Health Insurance Physicians (NASHIP) provide financial support for physicians and psychotherapists who conduct video consultations. In addition, there is a financial support of NASHIP for the authentication of new patients with a video consultation and a NASHIP technology and funding surcharge for each individual video consultation [12, 13].

Factors influencing the use of telemedicine have been identified the patient and physician level [14, 15]. By analysing data from the American Medical Association’s 2016 Physician Practice Benchmark Survey, Kane et al. showed the relevance of the size of the practice, with particularly large practices more likely to offer telemedicine services. They also found that speciality is an important correlate of telemedicine use [16]. For example, at the patient level, the utilization of e-health for healthcare was significantly more frequent among female patients [17]. With regard to patient age as a significant predictor, the existing results differ. In their research model, Hennemann et al. could not find any influence of the age of the health professionals on the acceptance of eHealth interventions, while Peine et al. found a negative correlation of health professional’s age with the perception of telemedicine’s significance [18, 19].

The impact of the COVID-19 pandemic on the use of telemedicine with a focus on different ambulatory disciplines from the perspective of the physicians has not been systematically studied. The main objective of this study was to evaluate the use of telemedicine procedures, the perceived benefits of using telemedicine and influencing factors, especially medical specialty, during the COVID-19 pandemic. Secondary objective was to describe potential barriers to the use of telemedicine.

## Methods

### Design

Data analysed for this study were collected in the second wave of the series of anonymous, cross-sectional online surveys of the COVID-GAMS study (The COVID-19 Crisis and its impact on the German ambulatory sector—the physicians’ view; BMBF, funding no. 01KI2099) The first wave was conducted in July–September 2020, and the second in November/December 2020. A next wave is planned for September 2021. The instruments for data collection on the use of telemedicine, perceived barriers to the use, and perceived benefits among physician working in outpatient care were developed by the COVID-GAMS Study based on literature review, previous instruments and informed by representatives of the target groups. The questionnaire was checked for comprehensibility by scientists and ambulatory physicians not involved in the study. The wording of the questions is presented in the supplementary file (Table S1 and S2).

### Participants and recruitment

The present study includes data from 16 November 2020 to 1 January 2021. A total of 18,000 outpatient physicians (6500 general practitioners; 1000 cardiologists; 500 gastroenterologists; 2000 paediatricians; 2000 gynaecologists; 2000 ear, nose, and throat [ENT] specialists and 4000 dentists) were randomly selected from the NASHIP physicians’ directory. They were contacted by fax and e-mail and invited to participate in the anonymous survey. To increase the response rate, three reminders at intervals of 2 weeks were sent (the first by fax, the second and third one by e-mail). Moreover, the professional associations were informed trough informal channels about the study and invited to participate. A total sample of 1521 physicians could be used for the analysis. Owing to the different recruitment methods, a reliable calculation of the response rate is not possible. In this study, we analysed the responses of all specialties except dentists. The data from dentists (*n* = 251) were excluded from the telemedicine analyses presented here because it could not be assumed that telemedicine procedures were used in relevant cases owing to the nature of their work. The online survey was conducted anonymously and approved by the Ethics Committee of the University of Cologne. The study conditions had to be confirmed to participate in the survey. The survey could be stopped or interrupted at any time and continued later. Participation was voluntary and without expense allowance or remuneration.

### Measures

#### Dependent variables

Participants were asked whether and which form of telemedicine (telephone and/or video consultation) they used before the pandemic, in March/April 2020 and in November/December 2020. To differentiate possible effects according to the type of telecommunication, the use of telephone consultations (dichotomous expression [yes/no]) in November/December 2020 (model 1) and the use of video consultations (dichotomous expression [yes/no]) in November/December 2020 (model 2) were chosen as dependent variables. To assess the perceived benefits of telemedicine, this question was asked: “How do you assess the overall benefit of telemedicine?” The subjective assessment of the benefits of telemedicine procedures was captured on a 4-point Likert scale (very low/low/high/very high) and is explained by the independent variables in model 3. The following question was examined to assess the proportion of patients receiving telemedical care: How much of the total patient contact is currently via telephone, video, or digital applications?” This question had to be answered on an 11-item scale (intervals of 10%). Only those who reported the use of telephone and/or video consultations and/or other digital applications in November/December 2020 were surveyed. The reported percentage of telemedicine use was used as a further dependent variable in model 4 and model 5.

#### Independent variables

The predictors integrated in all models were the medical speciality (nominal: general medicine, cardiology, gastroenterology, paediatrics, gynaecology, ENT), practice location (nominal: rural community, town, mid-sized city, metropolitan area), age (interval: ≤30 years, 31–40 years, 41–50 years, 51–60 years, > 60 years), practice type (nominal: solo practice, group practice) and gender (nominal: male, female, diverse). Gender-diverse participants were excluded from the multivariate analysis owing to the small number (*n* = 3) and the subsequent statistical problems, and participants were differentiated into only male and female. Gender, age, and practice type were included as control variables, as some studies in the past have shown to influence the use of telemedicine of physicians or patients [16, 17, 19]. Therefore, these variables should be inserted to control confounding effects.

### Statistical analyses

The data were statistically analysed using descriptive and inferential statistics The categorical variables were quantitative expressed as numbers and percentages and handled as factor variables of Stata in the models. The coding of the individual variables is shown in the supplementary file (Table S1 and S2). The development of telemedicine use before the pandemic, in March/April 2020 and in November/December 2020 was assessed using the McNemar test.

Multivariate logistic regression analysis was conducted to assess the effect of the independent variables (speciality, practice location, age, practice type and gender) on the use of telephone consultations (model 1) and video consultations (model 2) in November/December 2020. The variance in the multivariate models 1 and 2 was assessed with Pseudo R-squared, and the influence of the independent variables was expressed using odds ratio (OR) estimates (95% confidence intervals [CI]). Multivariate linear regressions were conducted to examine the effect of independent variables (speciality, practice location, age, practice type and gender) on the assessment of the use of telemedicine (model 3) and the percentage of patient contact (model 4). Model 5 integrated the assessment of the benefits of telemedicine as an independent variable into model 4 to identify the effect of the perceived benefits of telemedicine. The variance in the multivariate models 3, 4 and 5 was assessed with adjusted R-squared, and the effect of the independent variables was expressed with regression coefficients (95% CI). *P* values ≤0.05 are considered statistically significant. In all five multivariate models, the individual specialities (cardiology, gastroenterology, paediatrics, gynaecology and ENT) were compared with general practice, and the individual practice locations (town, mid-sized city and metropolitan area) with rural community.

Missing data are described descriptively but not included in the calculations of the models. In the descriptive analysis, in addition to the variables included in the models 1–5 the frequency of use of telemedicine and the reasons given by physicians against using telemedicine were considered. Only those physicians who stated that they used telephone and/or video consultations at both timepoints—in March/April 2020 and November/December 2020—were asked about the frequency of use. Those who did not use telephone or video consultations were asked why they did not use telemedicine. All statistical analyses were performed using Stata software, version 16.1 (StataCorp LLC, College Station, TX).

## Results

### Demographic data and survey size

In total, the data of 1521 outpatient physicians was included in the analysis. Table 1 shows an overview of the demographic data. Most study participants (46.22%, *n* = 703) were aged between 51 and 60 years. Of the 1521 participants, 50.49% (*n* = 768) were female, 49.18% (*n* = 748) were male, 0.20% (*n* = 3) were gender diverse, and 0.13% (*n* = 2) did not provide any information on gender and therefore excluded from the multivariate analysis. The medical specialities were distributed as follows: general practitioners (49.38%, *n* = 751), gynaecology (17.23%, *n* = 262), paediatrics (14.66%, *n* = 223), ENT (07.89%, *n* = 120), gastroenterology (7.30%, *n* = 111) and cardiology (3.55%, *n* = 54). In terms of the practice type, 52.53% (*n* = 798) and 46.68% (*n* = 715) reported working in solo practice and group practice, respectively. In terms of regional location, most physicians (36.36%, *n* = 553) reported that their practice was in a major city with 100,000 or more inhabitants.

**Table 1:** Demographic characteristics of responders

Participants, N	All medical specialities	General medicine	Cardio-logy	Gastroen-terology	Pedia-trics	Gyneco-logy	ENT
	100% (1521)	49.38% (751)	3.55% (54)	7.30% (111)	14.66% (223)	17.23% (262)	7.89% (120)
**Age (years)**							
≤ 30	0.33% (5)	0.40% (3)	1.85% (1)	0.00% (0)	0.45% (1)	0.00% (0)	0.00% (0)
31–40	5.26% (80)	5.59% (42)	3.70% (2)	0.90% (1)	7.62% (17)	3.05% (8)	8.33% (10)
41–50	25.18% (383)	23.44% (176)	25.93% (14)	27.93% (31)	29.15% (65)	25.19% (66)	25.83% (383)
51–60	46.22% (703)	45.14% (339)	50.00% (27)	53.15% (59)	42.15% (94)	50.38% (132)	43.33% (52)
> 60	22.55% (343)	25.03% (188)	16.67% (9)	18.02% (20)	19.73% (44)	20.99% (55)	22.50% (27)
missing value	0.46% (7)	0.40% (3)	1.85% (1)	0.00% (0)	0.90% (2)	0.38% (1)	0.00% (0)
**Gender**							
female	50.49% (768)	47.54% (357)	18.52% (10)	18.92% (21)	56.95% (127)	78.24% (205)	40.00% (48)
male	49.18% (748)	52.33% (393)	79.63% (43)	80.18% (89)	42.15% (94)	21.76% (57)	60.00% (72)
diverse	0.20% (3)	0.00% (0)	0.00% (0)	0.90% (1)	0.90% (2)	0.00% (0)	0.00% (0)
missing value	0.13% (2)	0.13% (1)	1.85% (1)	0.00% (0)	0.00% (0)	0.00% (0)	0.00% (0)
**Practice type**							
solo practice	52.53% (799)	50.47% (379)	31.48% (17)	37.84% (42)	58.74% (131)	61.07% (160)	58.33% (70)
group practice	46.68% (711)	48.87% (367)	66.67% (36)	62.16% (69)	39.46% (88)	38.55% (101)	40.83% (49)
missing value	0.79% (12)	0.67% (5)	1.85% (1)	0.00% (0)	1.79% (4)	0.38% (1)	0.83% (1)
**Practice location**							
rural community (< 5000)	9.20% (140)	16.11% (121)	0.00% (0)	1.80% (2)	3.59% (8)	2.67% (7)	1.67% (2)
town (5000–19,999)	23.73% (361)	27.03% (203)	20.37% (11)	16.22% (18)	23.32% (52)	20.99% (55)	18.33% (22)
mid-sized city (20000–99,999)	30.11% (458)	25.30% (190)	40.74% (22)	36.04% (40)	32.74% (73)	31.30% (82)	42.50% (51)
metropolitan area (100,000 or more)	36.36% (553)	31.03% (233)	37.04% (20)	45.95% (51)	39.46% (88)	44.27% (116)	37.50% (45)
missing value	0.59% (9)	0.53% (4)	1.85% (1)	0.00% (0)	0.90% (2)	0.76% (2)	0.00% (0)

### Differences in telemedicine use according to medical speciality

**Fig. 1:**
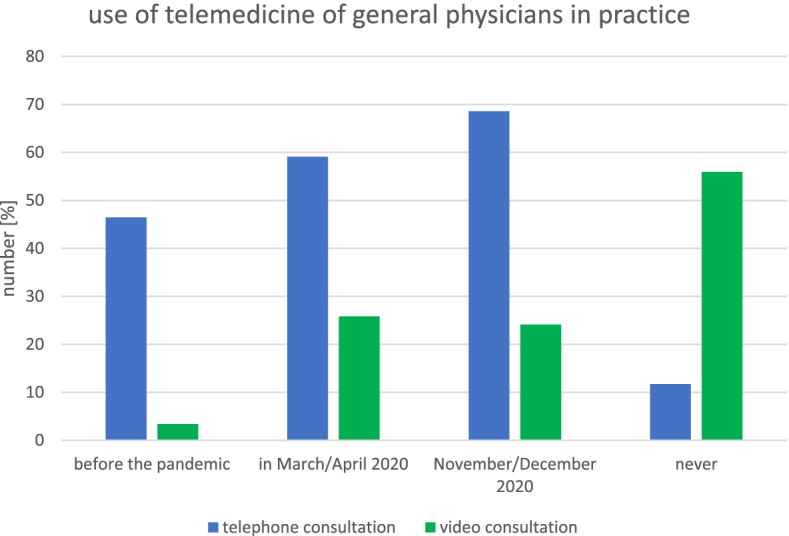
Use of telemedicine (telephone and video consultation) in general practice before the pandemic, in March/April 2020, in November/December 2020 and never

**Fig. 2:**
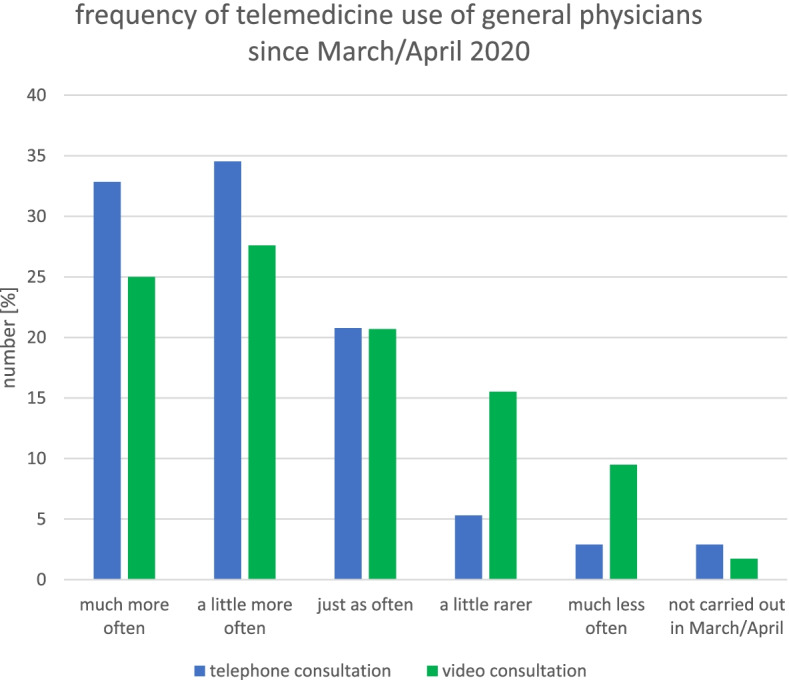
Change in the frequency of use of telemedicine since March/April 2020

Figure 1 shows the development of telemedicine in general practice before the pandemic, in March/April 2020 and November/December 2020. In total, 46.47% (*n* = 349) of the general practitioners surveyed said that they had used telephone consultation before the pandemic. For the period March/April 2020, 59.12% (*n* = 444) reported having used telephone consultation; this represents a significant increase from before the pandemic (McNemar, *p* = 0.00). The number of general practitioners using telephone consultation in November/December 2020 (68.58%, *n* = 515) was significantly higher than that in March/April 2020 (McNemar, p = 0.00). Overall, 11.72% (*n* = 88) of the general practitioners had not used telephone consultation at any time, and 5.59% (*n* = 42) did not report whether they used it. A comparison of the number of physicians using video consultation pre-pandemic (3.37%, *n* = 28) and in March/April 2020 (25.83%, *n* = 194) showed a significant difference (McNemar, p = 0.00). No significant difference was observed between March/April 2020 and November/December 2020 (24.10%, *n* = 181). The majority (55.93%, *n* = 420) reported not having used video consultation at any time, and 7.59% (*n* = 57) did not report whether they used it. In terms of frequency of use, most general practitioners reported using telephone consultations (34.54%, *n* = 136) and video consultations (27.00%, *n* = 32) a little more frequently than in March/April 2020 (Fig. 2). The main reasons given by physicians for not using telemedicine (16.54%, *n* = 252) are that the treatment is not equivalent to face-to-face contact (68.65%, *n* = 173), that patient demand is low (53.97%, n = 136), and that the organizational burden is too high (44.84%, *n* = 113).

If differentiated by medical speciality, the highest usage rate of telephone consultations in November/December 2020 was in general medicine (68.58%, *n* = 515), whereas cardiology shows the lowest usage rate (37.04%, *n* = 20; Table 2). Video consultation was most commonly used in paediatrics (30.94%, *n* = 69) and general medicine (24.10%, *n* = 181). The lowest usage rate of video consultation was also found in cardiology (12.96% *n* = 7; Table 2). In terms of benefit assessment, telemedicine was rated highest in general practice, lowest in ENT (Table 3). The most frequently reported percentage of telemedicine use in November/December 2020 in all specialities was 10%. The distribution of percentages varies by specialty. General practitioners, paediatricians and gynaecologists are represented in the higher percentages, cardiology and ENT in the lower ones (Table 4).

**Table 2:** Use of telephone and video consultation in November/December 2020 differentiated according to medical speciality

Medical speciality	Telephone consultation November/December 2020			Video consultation November/December 2020		
	yes	no	missing value	yes	no	missing value
General medicine 100% (751)	68.58% (515)	25.83% (194)	5.59% (42)	24.10% (181)	68.31 (513)	7.59% (57)
Cardiology 100% (54)	37.04% (20)	57.42% (31)	5.56% (3)	12.96% (7)	79.63% (43)	7.41% (4)
Gastroenterology 100% (111)	63.06% (70)	33.33% (37)	3.60% (4)	17.12% (19)	75.68% (84)	7.21% (8)
Paediatrics 100% (223)	64.57% (144)	31.39% (70)	4.04% (9)	30.94% (69)	63.68% (142)	5.38% (12)
Gynaecology 100% (262)	61.07% (160)	35.50% (93)	3.44% (9)	12.98% (34)	78.24% (205)	8.78% (23)
ENT 100% (120)	40.83% (49)	53.33% (64)	5.83% (7)	13.33% (16)	79.17% (95)	7.50% (9)

**Table 3:** Physicians’ assessment of the benefit of telemedicine differentiated according to medical speciality

Medical speciality	How do you assess the overall benefit of telemedicine?				
	very high	high	low	very low	missing value
General medicine 100% (751)	7.86% (59)	35.69% (268)	42.08% (316)	10.12% (76)	4.26% (32)
Cardiology 100% (54)	1.85% (1)	37.04% (20)	46.30% (25)	11.11% (6)	3.70% (2)
Gastroenterology 100% (111)	8.11% (9)	35.14% (39)	46.85% (52)	7.21% (8)	2.70% (3)
Paediatrics 100% (223)	7.62% (17)	30.49% (68)	50.22% (112)	8.07% (18)	3.59% (8)
Gynaecology 100% (262)	6.11% (16)	29.39% (77)	53.82% (141)	8.78% (23)	1.91% (5)
ENT 100% (120)	0.83% (1)	21.67% (26)	53.33% (64)	20.00% (24)	4.17% (5)

**Table 4:** Proportion of patient contact via telemedicine differentiated according to medical speciality

Medical specia-lity	How much of the total patient contact is currently (November/December 2020) via telephone, video or digital applications?											
	0%	10%	20%	30%	40%	50%	60%	70%	80%	90%	100%	missing
General medicine 100% (555)	3.24% (18)	50.09% (278)	28.11% (156)	10.63% (59)	3.78% (21)	1.98% (11)	0.36% (2)	0.72% (4)	0.36% (2)	0.00% (0)	0.36% (2)	0.36% (2)
Cardio-logy 100% (26)	11.54% (3)	76.92% (20)	11.54% (3)	0.00% (0)	0.00% (0)	0.00% (0)	0.00% (0)	0.00% (0)	0.00% (0)	0.00% (0)	0.00% (0)	0.00% (0)
Gastro-entero-logy 100% (74)	6.76% (5)	62.16% (46)	21.62% (16)	8.11% (6)	1.35% (1)	0.00% (0)	0.00% (0)	0.00% (0)	0.00% (0)	0.00% (0)	0.00% (0)	0.00% (0)
Paedia-trics 100% (157)	6.37% (10)	63.06% (99)	19.11% (30)	4.46% (7)	3.18% (5)	0.64% (1)	0.64% (1)	0.00% (0)	1.27% (2)	0.64% (1)	0.00% (0)	0.64% (1)
Gyneco-logy 100% (173)	5.20% (9)	79.19% (137)	10.98% (19)	3.47% (6)	0.58% (1)	0.00% (0)	0.00% (0)	0.00% (0)	0.00% (0)	0.00% (0)	0.00% (0)	0.58% (1)
ENT 100% (57)	19.30% (11)	70.18% (40)	10.53% (6)	0.00% (0)	0.00% (0)	0.00% (0)	0.00% (0)	0.00% (0)	0.00% (0)	0.00% (0)	0.00% (0)	0.00% (0)

### Predictors and multivariate models for the use of telemedicine

The potential influencing factors of the use of telephone consultation in November/December 2020 were identified in model 1 (logistic regression, Table 5). The disciplines ENT (OR 0.28, *p* = 0.00), cardiology (OR 0.23, *p* = 0.00), gynaecology (OR 0.53, *p* = 0.00) and paediatrics (OR 0.68, *p* = 0.03) showed a significantly lower association with the use of telephone consultation than general practice. Physicians working in practices in metropolitan areas (OR 1.59, *p* = 0.03) and mid-sized cities (OR 1.59, p = 0.03) showed a significantly higher association with the use of telephone consultations than those working in practices in rural areas. Gender also showed a significant association, with female physicians more likely to use telephone consultations than males (OR 1.48, *p* = 0.00). Regarding the use of video consultations, model 2 (logistic regression; Table 5) yielded the following results. General medicine showed a significantly higher association with the use of video consultations than gynaecology (OR 0.46, *p* = 0.01), ENT (OR 0.46, *p* = 0.01) and cardiology (OR 0.41, *p* = 0.03).

**Table 5:** Multivariate logistic regression analysis of predictors associated with telephone/video consultation

	**telephone consultation (model 1)**		**video consultation (model 2)**	
Number of obs.	1427		1392	
LR chi2 (11)	74.68		41.54	
Prob > chi	0.00		0.00	
Pseudo R-squared	0.04		0.03	
	**Multivariable OR (95% CI)**	***p*** **value**	**Multivariable OR (95% CI)**	***p*** **value**
**Speciality** (Reference: General medicine)				
Cardiology	0.23 (0.13–0.43)	0.00**	0.41 (0.18–0.94)	0.03*
Gastroenterology	0.70 (0.44–1.08)	0.11	0.59 (0.35–1.02)	0.06
Paediatrics	0.68 (0.48–0.96)	0.03*	1.30 (0.91–1.83)	0.16
Gynaecology	0.53 (0.38–0.74)	0.00**	0.46 (0.30–0.70)	0.00**
ENT	0.28 (0.18–0.42)	0.00**	0.46 (0.26–0.81)	0.01**
**Practice location** (Reference: rural community)				
Town	1.44 (0.93–2.22)	0.10	1.45 (0.87–2.43)	0.15
mid-sized city	1.59 (1.04–2.45)	0.03*	1.54 (0.93–2.57)	0.09
metropolitan area	1.59 (1.05–2.44)	0.03*	1.55 (0.94–2.55)	0.09
**Age**	0.93 (0.81–1.07)	0.29	0.90 (0.77–1.04)	0.16
**Practice type** (Reference: individual practice)	1.22 (0.96–1.53)	0.09	1.28 (0.99–1.66)	0.06
**Gender** (Reference: male)	1.48 (1.16–1.89)	0.00**	1.00 (0.77–1.31)	0.98

*p ≤ 0.05; **p ≤ 0.01

The overall assessment of the benefits of telemedicine was analysed in model 3 (linear regression; Table 6). The disciplines ENT (Coef − 0.40, *p* = 0.00) and gynaecology (Coef − 0.18, *p* = 0.00) showed a significantly lower correlation with benefit assessment than general practice. The metropolitan area (Coef 0.25, *p* = 0.00) was more highly correlated with the evaluation of the benefits of telemedicine than the rural area. The type of practice also showed a significant positive correlation (Coef 0.13, *p* = 0.00) with benefit assessment, with physicians in group practice being more likely to find telemedicine useful than those in solo practice. Female physicians were more likely to perceive benefits of telemedicine than male physicians, such that a positive correlation of gender (Coef 0.22, *p* = 0.00) was observed. Considering the proportion of total patient contact accomplished with telemedicine in model 4 (linear regression), the following results were obtained (Table 6). General practice showed a significantly higher correlation with the proportion of patient contact established via telemedicine than gynaecology (Coef − 0.71, *p* = 0.00), ENT (Coef − 0.89, *p* = 0.00), cardiology (Coef − 0.76, *p* = 0.00), gastroenterology (Coef − 0.45, *p* = 0.00) and paediatrics (Coef − 0.30, *p* = 0.00). Physicians with practices in metropolitan areas (Coef 0.35, *p* = 0.01) and in mid-sized cities (Coef 0.27, *p* = 0.05) showed a significantly higher correlation with the proportion of patient contact established via telemedicine than physicians with practices in rural areas. Gender also showed a significant correlation (Coef 0.17, *p* = 0.03), with female physicians being more likely to have telemedicine contacts than male physicians. In model 5 (linear regression) and model 4 the same statistical significance values were obtained for the medical speciality. In addition, the assessment of the benefits of telemedicine showed a highly significant correlation with the proportion of patient contact achieved through telemedicine (Coef 0.37, *p* = 0.00; Table 6).

**Table 6:** Multivariate linear regression analysis of predictors associated with the physicians’ assessment of the benefits of telemedicine and with the proportion of patient contact through telemedicine services

	**Physicians’ assessment of the benefits of telemedicine (model 3)**		**Proportion of patient contact (model 4)**		**Proportion of patient contact (model 5)**	
Number of obs.	1446		1026		1025	
F (11)	8.03		8.05		13.14	
Prob > F	0.00		0.00		0.00	
adj. R-squared	0.06		0.07		0.12	
	**Multivariable Coef. (95% CI)**	***p*** **value**	**Multivariable Coef. (95% CI)**	***p*** **value**	**Multivariable Coef. (95% CI)**	***p*** **value**
**Speciality (**Reference: General medicine)						
Cardiology	−0.12 (−0.33–0.10)	0.30	−0.76 (−1.21−−0.32)	0.00**	−0.77 (−1.21−−0.34)	0.00**
Gastroenterology	0.02 (−0.13–0.18)	0.78	−0.44 (−0.72−−1.16)	0.00**	−0.45 (− 0.72−−1.17)	0.00**
Paediatrics	0.09 (− 0.21–0.02)	0.14	− 0.30 (− 0.51−− 0.10)	0.00**	− 0.29 (− 0.49−− 0.09)	0.01**
Gynaecology	− 0.18 (− 0.29−− 0.07)	0.00**	− 0.71 (− 0.92−− 0.51)	0.00**	−0.66 (− 0.86−− 0.46)	0.00**
ENT	−0.40 (− 0.55−− 0.25)	0.00**	−0.89 (− 1.20−− 0.58)	0.00**	−0.76 (− 1.06−− 0.45)	0.00**
**Practice location (**Reference: rural community)						
town	0.12 (−0.03–0.27)	0.12	0.19 (−0.08–0.47)	0.17	0.16 (−0.10–0.43)	0.23
mid-sized city	0.14 (−0.01–0.29)	0.07	0.27 (0.00–0.55)	0.05*	0.22 (−0.04–0.48)	0.10
metropolitan area	0.25 (0.10–0.39)	0.00**	0.35 (0.08–0.61)	0.01**	0.25 (−0.01–0.51)	0.06
**Age**	−0.03 (− 0.08–0.02)	0.23	0.07 (−0.01–0.16)	0.09	0.07 (−0.01–0.16)	0.08
**Practice type** (Reference: individual practice)	0.13 (0.06–0.21)	0.00**	0.09 (−0.05–0.23)	0.20	0.05 (−0.09–0.19)	0.48
**Gender** (Reference: male)	0.22 (0.14–0.30)	0.00**	0.17 (0.02–0.31)	0.03*	0.09 (−0.05–0.23)	0.22
**Physician’s assessment of the benefits**					0.37 (0.28–0.47)	0.00**

*p ≤ 0.05; **p ≤ 0.01

## Discussion

In comparison with general medicine, all other specialities reported a significantly lower use of telephone consultations, except for gastroenterology, where the effect was not significant. All specialities except gastroenterology and paediatrics also reported a significantly lower use of video consultations than general practitioners. Gastroenterologists and gynaecologists assessed the benefits of telemedicine to be significantly lower than general practitioners. The proportion of patient contact through telemedicine is significantly higher in general medicine than in all other specialities. A significantly higher use and positive assessment of telemedicine was observed in metropolitan areas than in rural areas, with all dependent variables except video consultation. The effect of gender showed the same characteristics, with female physicians reporting higher use of telephone consultations, higher assessment of telemedicine and higher portion of patient contact with telemedicine than male physicians. Physicians in group practice rated the benefits of telemedicine significantly higher than physicians in solo practice. Finally, the assessment of the benefits of telemedicine showed a high significant correlation with the proportion of patient care provided using telemedicine.

The most common age group in the study was 51–60 years, which is representative of the average age of physicians in NASHIP-accredited medical care. The average age of physicians in Germany in 2020 was 54.2 years [20]. The proportion of female physicians in our study was 50.49%, which is comparable to the proportion of female physicians in Germany in 2020 (48.9%) according to the NASHIP. In our study, general practitioners accounted for 49.38% of the participants (NASHIP 55.01%), cardiologists for 3.55% (NASHIP 3.48%), gastroenterologists for 7.30% (NASHIP 2.17%), paediatricians for 14.66% (8.04%), gynaecologists for 17.23% (12.7%) and ENT specialists for 7.89% (4.59%) [20]. The distribution shows slight deviations between our study and the NASHIP data, which may be attributed to a higher number of specialities included in the NASHIP analysis. In our study, 52.47% of physicians work in solo practice, which is representative for Germany with 58% solo practices according to the NASHIP [21].

The observed differences by medical speciality are also reflected in a recent analysis conducted by the NASHIP based on billing data. According to this analysis, psychotherapists use video consultations most frequently, followed by general practitioners and paediatricians. These findings correspond to our results [22]. A policy brief to the use of digital health tools in Europe indicates different areas used digital health tools during the pandemic, such as communication and information, surveillance and monitoring, and remote consultations [23]. In another investigation of the use of telemedicine differentiated by specialist groups, different modalities of telemedicine were examined. Cardiologists represented the specialist group with the highest use of remote patient monitoring [16]. The remote monitoring data acquisition system consists of different sensors or devices with embedded sensors with data transmission capability wireless [24]: This may explain why cardiologists in our study were the least likely of all specialists to report using telephone and video consultations. Thus, different modalities of telemedicine are used in different specialities. There is a different need for telemedicine and a perception differentiated according to medical speciality is important. In the present study, the practice location was positively associated in model 1 and positive correlated in models 3 and 4 with the metropolitan area, with no significant association in model 2. In contrast, the multivariate model of Kane et al. shows that a practice in a non-metropolitan area is more likely to be associated with the use of videoconferencing than that in a metropolitan area [16]. This may be explained by the fact that, according to our study, the use of telemedicine (telephone and video consultations) has increased strongly overall owing to the COVID-19 pandemic. Whether the use of video consultations has increased more in the metropolitan area than in the rural area requires further research. The influence of gender may be explained by the different communication styles of men and women. According to Weisman et al., female physicians are generally more interpersonally oriented [25]. They are more interested in patient involvement and partnership [26, 27]. In addition, gender differences are more pronounced among health care providers than among patients [28]. This suggests that situation-specific considerations, such as perceived role, may override the gender-specific behaviours when actors adapt their communication to different situations [29]. These factors may be why female physicians are more likely to use telemedicine procedures. The factor of practice type was also distinguished in the multivariate model of Kane et al. A larger practice size is associated with a greater likelihood of using each measure of telemedicine [16]. In our study, however, a correlation with practice type was found only in the evaluation of the benefits of telemedicine (model 3). The fact that no significant correlation was found between practice type and the use of telephone and video consultations (models 1 and 2) may be related to the general increase in the use of telemedicine by all practices through the COVID-19 pandemic. The research models mentioned below show that the use of telemedicine depends on physicians’ assessment of it, as observed in our model 5. The model by Kuo et al. shows that physicians’ attitude, subjective norm, and perceived behavioural control are positively associated with the behavioural intention to use telemedicine [30]. Another study attempted to correlate physicians’ satisfaction with the adoption and use of telemedicine services using the technology acceptance model [31]. Perceived ease of use and perceived usefulness of telemedicine services were found to influence physicians’ behavioural intentions [31]. In another multivariate regression model, it was shown that perceived usefulness of telemedicine is influenced by previous experience with telemedicine, the quality of clinical practice and patient health [32].

To further establish telemedicine, it is also important to explore the background factors that motivate patients to either use or not use telemedicine. Our survey revealed that a barrier among physicians who do not use telemedicine is that patient demand is low. In their research in China during the COVID-19 pandemic, Li et al. found that perceived behavioural control and perceived severity of illness are the most important determinants of intention to use the online inquiry services of Internet hospitals [33].

Looking at the entire field of medical specialities, the lack of randomized controlled trials in the research area of telemedicine is criticized internationally [34]. Concerns about data protection, lack of interoperability, major differences in regional funding, a lack of proof of benefit for inclusion in the statutory health insurance (NASHIP) benefits catalogue and thus a lack of possibility of billing for telemedicine services constitute some of the barriers to establishing telemedicine in Germany [10, 11]. Adequate funding of telemedicine is a problem internationally. Flodgren et al. argue that the costs to the health system and the effectiveness of telemedicine are unclear for many programmes due to limited data [35]. In addition, there is a lack of training for physicians, practice assistants and nursing staff in telemedicine technology [36, 37]. That training has a positive impact is shown by a cross-sectional survey of Donelan et al. In 15 clinical departments, physicians were trained for 1 year on how to conduct virtual video visits as part of the Massachusetts General Hospital (MGH) TeleHealth programme. 59.0% of physicians reported that there was no difference in the “overall quality of the visit” between the virtual visit and the office visit [38]. These could be explanations for the barriers mentioned by the physicians in our study, that treatment via telemedicine is not equivalent to face-to-face contact and the organisational burden is too high. Therefore, initial funding, process restructuring and employee flexibility are required [39].

### Limitations

The study may have certain limitations. First, although the inclusion of the physicians was anonymous and random, an influence on the results in the sense of social desirability cannot be excluded. It is possible that predominantly physicians who show a high level of commitment answered the questionnaire and that this group of physicians answered differently than the average, leading to a selection bias.

Second, the models 1–4 showed a low variance (0.04, 0.03, 0.06 and 0.07, respectively), which may be explained by the suppression effect of the personal identity in the regression equations. Model 5, which integrates the assessment of benefits, showed a higher variance (0.12) than model 4. These models and previous studies suggest that physicians’ intention to use telemedicine can be better predicted when their self-perception as telemedicine users is considered [30, 31]. Because the assessment of benefits in our questionnaire was only for telemedicine as a whole and did not differentiate between video and telephone consultations, the physicians’ self-perception could not be integrated into model 1 and 2. There are further factors, which were not considered here, have an influence on the perceived usefulness of telemedicine by physicians [32].

Furthermore, comparability of the models was difficult owing to different numbers of observations. Model 1–3 presented a significant higher number of observations (*N* = 1427, 1392 and 1446, respectively) than model 4 (*N* = 1026) and 5 (*N* = 1025). Finally, we were not able to estimate how frequently telephone and video consultation were used in each case. The number of physicians using telephone and video consultation in their practice was determined, but not the frequency of the individual functions. The frequency of the use was only determined overall for telemedicine.

## Conclusions

The COVID-19 pandemic has had an immediate impact on physician behaviour, which is also evident in physicians’ subjective assessments and in healthcare data. With the COVID-19 pandemic, the use of telemedicine among outpatient physicians has increased significantly. The extent to which sustainable changes are on the horizon here, or whether there will be a return to established patterns, will be seen once the pandemic has subsided. Further research is needed in the context of the different extents of the use of telemedicine procedures and perceived benefits of the use of telemedicine among medical specialities and which factors determine the differences between men and women, rural and metropolitan location of the practice, individual and group practice. The physician’s and patient’s barriers that have prevented telephone and video consultations from gaining acceptance by physicians needed to be reduced. To intervene barriers and improve the assessment of telemedicine use, training for both physicians and patients could be offered. At the same time, bureaucratic barriers could be simplified. To ensure that the advantages of telemedicine can be used, it is necessary to learn more about the evaluation of telemedicine from the patient’s point of view as well. If it becomes clear that patients benefit from digital offerings, the barriers mentioned by physicians must be removed.

## Supplementary Information

**Additional file 1: Table S1.** Questionnaire (German, original version, Excerpt). **Table S2**. Questionnaire (English translation, Excerpt).

## Data Availability

The dataset generated and analysed during the current study are available from the corresponding author on reasonable request.

## References

[1] Bundesärztekammer (AG-Telemedizin) (2015). Telemedizinische Methoden in der Patientenversorgung –Begriffliche Verortung.

[2] Wax RS, Christian MD (2020). Practical recommendations for critical care and anesthesiology teams caring for novel coronavirus (2019-nCoV) patients. Can J Anesth/J Can Anesth.

[3] Lurie N, Carr BG (2018). The Role of Telehealth in the Medical Response to Disasters. JAMA Intern Med.

[4] Doarn CR, Merrell RC (2014). Telemedicine and e-health in disaster response. Telemed J E Health.

[5] Hollander JE, Carr BG (2020). Virtually Perfect? Telemedicine for Covid-19. N Engl J Med.

[6] Klein BC, Busis NA (2020). COVID-19 is catalyzing the adoption of teleneurology. Neurology.

[7] Mihalj M, Carrel T, Gregoric ID, Andereggen L, Zinn PO, Doll D (2020). Telemedicine for preoperative assessment during a COVID-19 pandemic: Recommendations for clinical care. Best Pract Res Clin Anaesthesiol.

[8] Center of Connected Health Policy (2021). COVID-19 telehealth coverage policies.

[9] Smith AC, Thomas E, Snoswell CL, Haydon H, Mehrotra A, Clemensen J, Caffery LJ (2020). Telehealth for global emergencies: Implications for coronavirus disease 2019 (COVID-19). J Telemed Telecare.

[10] Beckers R (2017). Status quo und Potenzial der Telemedizin in Deutschland. Anasthesiol Intensivmed Notfallmed Schmerzther.

[11] Marx G, Beckers R (2015). Telemedizin in Deutschland Bundesgesundheitsbla..

[12] Haserück A (2020). Schub durch Coronapandemie. Deutsches Ärzteblatt PP.

[13] Kassenärztliche Bundesvereinigung (KBV). Videosprechstunde. 2020. https://www.kbv.de/html/videosprechstunde.php. Accessed 5 Nov 2020.

[14] Scott Kruse C, Karem P, Shifflett K, Vegi L, Ravi K, Brooks M (2016). Evaluating barriers to adopting telemedicine worldwide: A systematic review. J Telemed Telecare.

[15] Almathami HKY, Win KT, Vlahu-Gjorgievska E (2020). Barriers and Facilitators That Influence Telemedicine-Based, Real-Time, Online Consultation at Patients’ Homes: Systematic Literature Review. J Med Internet Res.

[16] Kane CK, Gillis K (2018). The Use Of Telemedicine By Physicians: Still The Exception Rather Than The Rule. Health Aff (Millwood).

[17] Kontos E, Blake KD, Chou W-YS, Prestin A (2014). Predictors of eHealth usage: insights on the digital divide from the Health Information National Trends Survey 2012. J Med Internet Res.

[18] Hennemann S, Beutel ME, Zwerenz R (2017). Ready for eHealth? Health Professionals' Acceptance and Adoption of eHealth Interventions in Inpatient Routine Care. J Health Commun.

[19] Peine A, Paffenholz P, Martin L, Dohmen S, Marx G, Loosen SH (2020). Telemedicine in Germany During the COVID-19 Pandemic: Multi-Professional National Survey. J Med Internet Res.

[20] Kassenärztliche Bundesvereinigung (KBV) (2020). Statistische Informationen aus dem Bundesarztregister.

[21] Kassenärztliche Bundesvereinigung (KBV). Kooperationen. 2020. https://www.kbv.de/html/14365.php. Accessed 23 Apr 2021.

[22] Kassenärztliche Bundesvereinigung (KBV) (2021). Praxisnachrichten 04.02.2021: Immer mehr Praxen greifen zur Kamera - Zahl der Videosprechstunden auf über eine Million gestiegen.

[23] Fahy N, Williams GA, Habicht T, Köhler K, Jormanainen V, Satokangas M (2021). Use of digital health tools in Europe: before, during and after COVID-19: Policy Brief.

[24] Malasinghe LP, Ramzan N, Dahal K (2017). Remote patient monitoring: a comprehensive study. J Ambient Intell Human Comput.

[25] Weisman CS, Teitelbaum MA (1985). Physician gender and the physician-patient relationship: Recent evidence and relevant questions. Soc Sci Med.

[26] Beisecker AE, Murden RA, Moore WP, Graham D, Nelmig L (1996). Attitudes of medical students and primary care physicians regarding input of older and younger patients in medical decisions. Med Care.

[27] Krupat E, Rosenkranz SL, Yeager CM, Barnard K, Putnam SM, Inui TS (2000). The practice orientations of physicians and patients: the effect of doctor–patient congruence on satisfaction. Patient Educ Couns.

[28] Hall JA, Roter DL (1998). Medical communication and gender: a summary of research. J Gend Specif Med.

[29] Street RL (2002). Gender differences in health care provider–patient communication: are they due to style, stereotypes, or accommodation?. Patient Educ Couns.

[30] Kuo K-M, Talley PC, Lee C-M, Yen Y-C (2015). The influence of telemedicine experience on physicians' perceptions regarding adoption. Tlemed J E Health.

[31] Kissi J, Dai B, Dogbe CS, Banahene J, Ernest O (2020). Predictive factors of physicians' satisfaction with telemedicine services acceptance. Health Informatics J.

[32] Ruiz Morilla MD, Sans M, Casasa A, Giménez N (2017). Implementing technology in healthcare: insights from physicians. BMC Med Inform Decis Mak.

[33] Li D, Hu Y, Pfaff H, Wang L, Deng L, Lu C (2020). Determinants of Patients' Intention to Use the Online Inquiry Services Provided by Internet Hospitals: Empirical Evidence From China. J Med Internet Res.

[34] Ekeland AG, Bowes A, Flottorp S (2010). Effectiveness of telemedicine: a systematic review of reviews. Int J Med Inform.

[35] Flodgren G, Rachas A, Farmer AJ, Inzitari M, Shepperd S. Interactive telemedicine: effects on professional practice and health care outcomes. Cochrane Database Syst Rev. 2015:CD002098. 10.1002/14651858.CD002098.pub2. 10.1002/14651858.CD002098.pub2 PMC6473731 26343551

[36] Brauns H-J, Loos W, Telemedizin in Deutschland. (2015). Stand - Hemmnisse - Perspektiven. Bundesgesundheitsblatt Gesundheitsforschung Gesundheitsschutz.

[37] Lyngstad M, Hofoss D, Grimsmo A, Hellesø R (2015). Predictors for assessing electronic messaging between nurses and general practitioners as a useful tool for communication in home health care services: a cross-sectional study. J Med Internet Res.

[38] Donelan K, Barreto EA, Sossong S, Michael C, Estrada JJ, Cohen AB (2019). Patient and clinician experiences with telehealth for patient follow-up care. Am J Manag Care.

[39] Augustin M, Wimmer J, Biedermann T, Blaga R, Dierks C, Djamei V (2018). Praxis der Teledermatologie. J Dtsch Dermatol Ges.

