# Supplementary material for: Use of telemedicine in the outpatient sector during the COVID-19 pandemic: a cross-sectional survey of German physicians
Source: BMC Prim Care. 2022 Apr 23;23:92. doi: 10.1186/s12875-022-01699-7 (PMC9034069; doi:10.1186/s12875-022-01699-7)
Supplement: Supplementary file 1 — Additional file 1: Table S1. Questionnaire (German, original version, Excerpt). Table S2. Questionnaire (English translation, Excerpt). [file 12875_2022_1699_MOESM1_ESM.docx]

**Table S1**. Questionnaire (German, original version, Excerpt)

| Welcher Fachrichtung gehören Sie an?  [medical speciality] | - Hausärztlich tätige/r Internist*in, FA für Allgemeinmedizin, Praktische/r Ärztin/Arzt [1] - Kardiologie [2] - Gastroenterologie [3] - Pädiatrie [4] - Gynäkologie [5] - HNO [6] - Zahnmedizin [7] |
| --- | --- |
| In welcher Praxisform arbeiten Sie?  [practice type] | - Einzelpraxis [1] - Berufsausübungsgemeinschaft (früher Gemeinschaftspraxis [2] |
| Wie groß ist der Ort, in dem Ihre Praxis liegt? [practice location] | - Landgemeinde (unter 5.000 Einwohner) [1] - Kleinstadt (5.000 – 19.999 Einwohner) [2] - Mittelstadt (20.000 – 99.999 Einwohner) [3] - Großstadt (100.000 Einwohner oder mehr) [4] |
| Wie alt sind Sie?  [age] | - unter 30 Jahre [1] - 31 bis 40 Jahre [2] - 41 bis 50 Jahre [3] - 51 bis 60 Jahre [4] - über 60 Jahre [5] |
| Welchem Geschlecht fühlen Sie sich zugehörig? [gender] | - männlich [1] - weiblich [2] - divers [3] |
| Welche Form der Telemedizin wurde bzw. wird in Ihrer Praxis genutzt? | |
| Telefonsprechstunde [telephone consultation]   - vor der Pandemie - im März/April - aktuell - nie | - ja [1]; nein [2] - ja [1]; nein [2] - ja [1]; nein [2] - ja [1]; nein [2] |
| Videosprechstunde [video consultation]   - vor der Pandemie - im März/April - aktuell - nie | - ja [1]; nein [2] - ja [1]; nein [2] - ja [1]; nein [2] - ja [1]; nein [2] |
| Warum wird in Ihrer Praxis keine Telemedizin genutzt? (barriers)   - Telemedizin ermöglicht keine ausreichende/gleichwertige Behandlung zum persönlichen Kontakt. - Es gibt keine/eine zu geringe Nachfrage nach Telemedizin durch die Patient*innen. - Es bestehen Datenschutzbedenken. - Die Anwendung von Telemedizin ist wirtschaftlich nicht lohnend. - Telemedizin erfordert einen zu hohen organisatorischen Aufwand. - Ich fühle mich bei der Nutzung von Telemedizin unwohl. | - ja [1]; nein [2] - ja [1]; nein [2] - ja [1]; nein [2] - ja [1]; nein [2] - ja [1]; nein [2] - ja [1]; nein [2] |
| Wie hat sich Ihre Nutzung von Telemedizin seit März/April (Zeitpunkt des ersten Lockdowns) bis heute verändert? | - viel häufiger [1] - etwas häufiger [2] - genauso oft [3] - etwas seltener [4] - viel seltener [5] - habe ich im März/April nicht durchgeführt [6] |
| Wie schätzen Sie den Nutzen von Telemedizin insgesamt ein? [physicians’assessment of the benefits of telemedicine] | - sehr hoch [1] - hoch [2] - gering [3] - sehr gering [4] |
| Wie viel Prozent des Patientenkontakts insgesamt entfallen aktuell auf Kontakt über Telefon, Video oder andere digitale Anwendungen?  [propotion of patient contact] | - 0% [1] - 10% [2] - 20% [3] - 30% [4] - 40% [5] - 50% [6] - 60% [7] - 70% [8] - 80% [9] - 90% [10] - 100% [11] |

**Table S2**. Questionnaire (English translation, Excerpt)

| Which medical specialty do you belong to? [medical speciality] | - General medicine [1] - Cardiology [2] - Gastroenterology [3] - Paediatrics [4] - Gynaecology [5] - ENT [6] - Dentistry [7] |
| --- | --- |
| What type of practice do you work in?  [practice type] | - Solo practice [1] - Group practice [2] |
| How big is the town where your practice is located?  [practice location] | - Rural community (under 5.000 inhabitants) [1] - Town (5.000 – 19.999 inhabitants) [2] - Mid-sized city (20.000 – 99.999 inhabitants) [3] - Metropolitan area (100.000 inhabitants or more) [4] |
| How old are you?  [age] | - under 30 years [1] - 31 to 40 years [2] - 41 to 50 years [3] - 51 to 60 years [4] - over 60 years [5] |
| Which gender do you feel you belong to? [gender] | - male [1] - female [2] - diverse [3] |
| What kind of telemedicine has been or is being used in your practice? | |
| Telephone consultation [telephone consultation]   - before the pandemic - in March/April - at this time - never | - yes [1]; no [2] - yes [1]; no [2] - yes [1]; no [2] - yes [1]; no [2] |
| Video consultation [video consultation]   - before the pandemic - in March/April - at this time - never | - yes [1]; no [2] - yes [1]; no [2] - yes [1]; no [2] - yes [1]; no [2] |
| Why is telemedicine not used in your practice?? (barriers)   - Telemedicine does not provide sufficient/equivalent treatment to face-to-face contact. - There is no/too little demand for telemedicine from patients. - There are data protection concerns. - The use of telemedicine is not economically worthwhile. - Telemedicine requires too much organisational effort. - I feel uncomfortable using telemedicine. | - yes [1]; no [2] - yes [1]; no [2] - yes [1]; no [2] - yes [1]; no [2] - yes [1]; no [2] - yes [1]; no [2] |
| How has your use of telemedicine changed from March/April (the time of the first lockdown) until today? | - Much more often [1] - a little more often [2] - just as often [3] - a little rarer [4] - much less often [5] - not carried out in March/April [6] |
| How do you assess the overall benefit of telemedicine? [physicians’ assessment of the benefits of telemedicine] | - very high [1] - high [2] - low [3] - very low [4] |
| How much of the total patient contact is currently via telephone, video, or digital applications?  [proportion of patient contact] | - 0% [1] - 10% [2] - 20% [3] - 30% [4] - 40% [5] - 50% [6] - 60% [7] - 70% [8] - 80% [9] - 90% [10] - 100% [11] |
